# GENLIB: an R package for the analysis of genealogical data

**DOI:** 10.1186/s12859-015-0581-5

**Published:** 2015-05-15

**Authors:** Héloïse Gauvin, Jean-François Lefebvre, Claudia Moreau, Eve-Marie Lavoie, Damian Labuda, Hélène Vézina, Marie-Hélène Roy-Gagnon

**Affiliations:** 1 Département de médecine sociale et préventive, Université de Montréal, Montréal, Québec Canada; 2 Centre de recherche, Centre hospitalier universitaire Sainte-Justine, Montréal, Québec Canada; 3 BALSAC Project, Université du Québec à Chicoutimi, Chicoutimi, Québec Canada; 4 Département de pédiatrie, Université de Montréal, Montréal, Québec Canada; 5 School of Epidemiology, Public Health and Preventive Medicine, Faculty of Medicine, University of Ottawa, 600 Peter Morand Cres, Room 101E, Ottawa, ON K1G 5Z3 Canada

**Keywords:** Genealogical data, Founder populations, Software, Historical demography, Kinship, Inbreeding, Genetics, Gene-dropping simulations

## Abstract

**Background:**

Founder populations have an important role in the study of genetic diseases. Access to detailed genealogical records is often one of their advantages. These genealogical data provide unique information for researchers in evolutionary and population genetics, demography and genetic epidemiology. However, analyzing large genealogical datasets requires specialized methods and software. The GENLIB software was developed to study the large genealogies of the French Canadian population of Quebec, Canada. These genealogies are accessible through the BALSAC database, which contains over 3 million records covering the whole province of Quebec over four centuries. Using this resource, extended pedigrees of up to 17 generations can be constructed from a sample of present-day individuals.

**Results:**

We have extended and implemented GENLIB as a package in the R environment for statistical computing and graphics, thus allowing optimal flexibility for users. The GENLIB package includes basic functions to manage genealogical data allowing, for example, extraction of a part of a genealogy or selection of specific individuals. There are also many functions providing information to describe the size and complexity of genealogies as well as functions to compute standard measures such as kinship, inbreeding and genetic contribution. GENLIB also includes functions for gene-dropping simulations.

The goal of this paper is to present the full functionalities of GENLIB. We used a sample of 140 individuals from the province of Quebec (Canada) to demonstrate GENLIB’s functions. Ascending genealogies for these individuals were reconstructed using BALSAC, yielding a large pedigree of 41,523 individuals. Using GENLIB’s functions, we provide a detailed description of these genealogical data in terms of completeness, genetic contribution of founders, relatedness, inbreeding and the overall complexity of the genealogical tree. We also present gene-dropping simulations based on the whole genealogy to investigate identical-by-descent sharing of alleles and chromosomal segments of different lengths and estimate probabilities of identical-by-descent sharing.

**Conclusions:**

The R package GENLIB provides a user friendly and flexible environment to analyze extensive genealogical data, allowing an efficient and easy integration of different types of data, analytical methods and additional developments and making this tool ideal for genealogical analysis.

**Electronic supplementary material:**

The online version of this article (doi:10.1186/s12859-015-0581-5) contains supplementary material, which is available to authorized users.

## Background

Studying founder or isolated populations is a major asset in reducing or taking into account the heterogeneity of genetic and environmental factors involved in complex diseases. Indeed, potential advantages of founder populations include greater genetic and environmental homogeneity and in some instances, the availability of extensive genealogical records [1]. Many founder populations across the world are currently studied, among others Iceland [2], the Amish [3-6], the Hutterites [7], the Mormons [8], Finland [9,10], Newfoundland [11], the Sardinians [12] and the French Canadian founder population of Quebec [13-15]. As the value of genealogical resources is increasingly recognized [16,17], efforts have been made to obtain extended genealogical information and build genealogical databases for use in genetic studies. To our knowledge, there are two available, freestanding packages for genealogical analysis, named PEDSYS [18] and PedHunter [19,20]. However, each lacks some functionality that we have found useful in our studies of the Quebec population and neither PEDSYS nor PedHunter can be used within a statistical computing environment such as R.

The BALSAC database is an example of a large genealogical database that has proven very valuable for genetic and demographic research in the Quebec founder population [21-23]. Briefly, the arrival of French settlers in the province of Quebec, Canada, at the beginning of the 17th century followed by the British Conquest about a century and a half later shaped the colonization history of Quebec and led to successive regional founder effects [21,24]. From ~8,500 settlers, the French speaking population now represents about 80% of the almost 8 million inhabitants of Quebec [25]. This fast population expansion and the regional founder effects have shaped genetic variation and population structure in Quebec. The related demographic information can be accessed using the BALSAC database, created 40 years ago, which now includes over 3 million vital event records providing information on 5 million individuals [26]. These records have been linked allowing for the reconstruction of ascending genealogies from present-day individuals going back over four centuries. The BALSAC data access policy and the procedures to request access to the BALSAC genealogical data can be found on the BALSAC website [26].

Specialized software is required in order to optimally exploit the full potential of these well preserved, exhaustive and detailed genealogical data. The S-Plus library of functions GENLIB was originally developed to work with the BALSAC database to manage, describe and visualize genealogies as well as perform allele-dropping simulations. The GENLIB S-Plus library has been used to study the selective advantage to be on an expanding wave front during a range expansion [14], the genetic contribution of non-French groups to the current population [13,27], population structure [28,29], drug intolerance for an inherited disorder in multigenerational pedigree [30] and patterns of genetic sharing relatively to expected sharing from genealogical information [31].

We have extended and implemented GENLIB in R to provide a freely accessible version of the software within a user-friendly and flexible environment and to allow a more efficient and easier integration of different types of data and analytical methods, also facilitating future developments. Such flexibility is especially important in the context of integrating large-scale genomic data and genealogical data. While other pedigree software exist, few are comprehensive, free and specific to extended genealogical databases. For example, the PedHunter [19,20] and PEDSYS [18] software include many functions to describe and analyze genealogical data but do not provide gene-dropping simulation functions. In this article, we present most of the functions in the GENLIB package. Using these functions, we perform a detailed description of a large genealogical corpus including 41,523 individuals provided as an example dataset in the R package. Then we show an example of gene-dropping simulations, a procedure in which hypothetical alleles, or chromosomal segments, are assigned to ancestors and dropped down the whole genealogical tree [32].

## Implementation

### Overview

GENLIB was originally written in the S programming language, integrating C++ functions to accelerate and extend S programming. We started with the same C++ functions in the translation of GENLIB into an R package and we extended both the C++ and R translation of the S code. Interaction between R and C++ is well supported and high quality R libraries are available to help implement such packages. The package is portable across Linux-like and Windows operating systems, and benefits from the advantages of the R environment, such as being open-source, available online under the GNU General Public License, and offering a wide range of complementary functions. All code has been tested using version 3.1.0 of R (http://cran.r-project.org). Version 1.0 of the GENLIB package presented in this paper includes over 40 functions. GENLIB is included in the CRAN packages repository (http://cran.r-project.org/web/packages) and is also available on the BALSAC website (http://balsac.uqac.ca). By installing the package, users also have access to the genealogical data described in this paper.

### Functions implemented

The starting point for any genealogical analysis in GENLIB is the creation of a genealogical object using the *gen.genealogy* function. The following input information needs to be provided in a matrix or data frame where each line corresponds to a subject: the subject identification number (ID), the subject’s father ID, the subject’s mother ID and subject’s sex (coded 1/2 for male/female). All values must be numerical. Functions from the package are grouped into 4 categories: i) management, ii) description and visualisation, iii) computation and iv) simulations. Table 1 provides an overview of most functions included in GENLIB.

**Table 1:** **Overview of GENLIB functions**

	Name	Use
**Functions to manage genealogical objects**		
	gen.genalogy	To create a basic genealogical object
	gen.lineages	To extract parental lineages from a genealogical object
	gen.branching	To extract a subset of a genealogical object
	gen.genout	To output a genealogical object as a data frame
	gen.founder, gen.half.founder, gen.pro, gen.parent, gen.sibship, gen.children, gen.findFounders, gen.findMRCA	To identify specific individuals (founder, half-founder, proband, parent, sibship, children, common founder, most recent common ancestor)
**Functions to describe…**		
	gen.nomen, gen.nowomen, gen.noind, gen.nochildren	…the number of men, women or individuals in a genealogy and number of kids an individual has
	gen.min, gen.mean, gen.max	…the minimal, mean or maximal generation at which an individual can be found in a genealogy (first generation is coded 0)
	gen.depth	…the number of generations in the genealogy
	gen.completeness	…genealogical data completeness
	gen.rec	…how many individuals within the specified individual group descend from each specified ancestor
	gen.occ	…how many different (but not mutually exclusive) paths link an ancestor to a descendant
	gen.meangendepth	…how much rooted are the genealogical lineages
	gen.implex	…the extent of pedigree collapse within an individual’s genealogy
	gen.findDistance	…distance between individuals through a specific ancestor
	gen.find.Min.Distance.MRCA	…the shortest distances between individuals
**Functions to plot…**		
	gen.graph	…the genealogy
**Functions to compute …**		
	gen.phi	…the kinship matrix at specified generations
	gen.f	…the inbreeding coefficients at specified generations
	gen.gc	…the genetic contribution of ancestors to individuals
**Gene-droppping simulation functions**		
	gen.simuProb	To compute the probability that individuals have 0, 1 or 2 copies of a disease allele knowing how many their ancestors had
	gen.simuSample, gen.simuSampleFreq	To obtain the number (frequencies) of disease alleles for each individual taking into account each ancestor’s carrier status
	gen.simuSet	As function gen.simuSample with option to customize transmission probabilities according to the parent’s and/or subject’s sex

Note: Additional functions (e.g., to calculate the variance associated with kinship and other measures) are available but not included in the table.

The first category includes functions allowing to track specific individuals (founders, siblings, probands, etc.) or to extract a part of a genealogy or a lineage. New functions to identify common founders and most recent common ancestors (MRCA) to a group of individuals have been implemented. In the second category, there are functions to count particular individuals, to compute the generational completeness and to describe the depth of the genealogical information and the number of paths linking two individuals. The completeness at a given generation (g) is the proportion of ancestors present in the genealogy (A_g_) relative to the maximum possible number of ancestors, i.e. assuming that all individuals at the previous generation have two known parents (2^g^) (see Table 2 for all formulas). When reproduction between two related individuals happens, the number of distinct ancestors in the family tree of their offspring is reduced. The implex index, which can also be computed with GENLIB, quantifies this collapsing phenomenon. In other words, the implex measures how much an individual’s ancestor tree deviates from a binary tree where the individual, his/her 2 parents, 4 grandparents, 8 great-grandparents and so on are all distinct individuals. Formally, the implex is calculated by taking the actual number of distinct ancestors (i.e., each ancestor is counted only once regardless of its number of occurrences) relative to the theoretical number of ancestors (2^g^) at a specified generation *g* [33]. Therefore the implex is also influenced by the information known about the ancestors and is always bounded by the completeness as an upper limit. In fact, to be informative, the implex should always be interpreted in the context of the completeness. Finally, the mean genealogical depth can be computed to get the expected generation where the founders can be found [33,34]. This value reflects the amount of information available in the genealogy as it is the average length of each lineage. In addition to characterizing genealogies, GENLIB provides a function to plot the genealogy (see Additional files 1 and 2: Figures S1 and S2 for examples of graphs).

**Table 2:** **Formulas of genealogical measures in GENLIB**

Completeness	${C}_g = \frac{A_g}{2^g}$	*A*_*g*_ : number of known ancestors at generation *g F*_*g*_ : number of founders at generation *g T*_*g*_ : number of expected ancestors at generation *g* (*=2* ^*g*^) *N*_*f*_ : generation of founder *f* (summation over all generations *g* or over all founders *f*)
Implex index	${I}_g = \frac{Distinct\ {A}_g}{2^g}$	
Mean genealogical depth	$D={\displaystyle \sum_gg\frac{F_g}{T_g}={\displaystyle \sum_f{N}_f}\frac{1}{2^{N_f}}}$	
Variance of mean genealogical depth	${\displaystyle \sum_g}g2\frac{F_g}{T_g}-D2$	
Kinship	${\varphi}_{ij} = {\displaystyle \sum_a}\left(1+{f}_a\right){\frac{1}{2}}^{g_{ai}+{g}_{aj}+1}$	*g*_*ai*_ (*g*_*aj*_) : number of generations between subject *i* (*j*) and ancestor *a*, common to *i* and *j* (summation over all ancestors *a*)
Inbreeding	*f*_*i*_ = *φ*_*kl*_	*k*, *l* : the parents of *i*
Genetic Contribution	$GC\left(s,a\right)={\displaystyle \sum_p}{\left(\frac{1}{2}\right)}^{g_p}$	*g*_*p*_ : number of generations between subject *s* and ancestor *a* through path *p* (summation over all possible paths *p*)

The third category of functions includes demogenetic functions. It is possible to compute the kinship and inbreeding coefficients [35-37] and the genetic contribution of an ancestor to its descendants [38,39] (see Table 2 for formulas). For kinship calculations that can be computationally intensive with GENLIB, the kinship function allows multi-threading. We compared kinship computation times between GENLIB and PedHunter by calculating kinship coefficients for all pairs of 140 probands using a six-core processor running at 2.667 GHz with 12 GB of RAM. The same results were obtained in 2 minutes with the multi-threading option in GENLIB and in 1 minute with PedHunter (excluding the preliminary data re-formatting steps required by PedHunter). The genetic contribution is the calculation of an ancestor’s contribution to the genetic makeup of an individual and this is obtained by summing up the probabilities of transmission over all genealogical paths connecting an ancestor and its descendant. Like other functions in the package, the computation of genetic contribution is customizable, meaning that it is possible to modify the inheritance pattern, which assumes that regardless the sex of parents and child, half the genetic material is transmitted.

Lastly, GENLIB provides functions to perform gene-dropping simulations. Other software implementing gene-dropping simulations (such as Mendel [40]), were conceived with a focus on whole pedigrees as opposed to reconstructed ascending genealogies from large founder populations, resulting in different functionalities and usage compared to GENLIB such as, for example, the ability to easily restrict simulations to specific individuals or ancestors. In GENLIB, different functions and their options allow to simulate genotype data for specific individuals in the genealogy based on a provided genealogical structure and according to the number of alleles carried by specific ancestors. Briefly, genotypes can be assigned to selected ancestors and segregated down the genealogy paths [32]. We added functionality to the gene-dropping simulations by implementing a segment-dropping option where the user is able to specify a recombination probability, i.e., a chance that the segment is not passed down as a whole. An option to specify the survival probability of homozygote carriers of a deleterious allele or regions was also added. An interesting application of the simulation functions is to estimate transmission probabilities for alleles or segments from ancestors to descendants, which can be computed quickly for simple relationships but can be difficult to obtain for complex genealogical structures.

### Datasets and simulations

Two genealogical datasets are provided with the package, one is a fictive example of genealogical data and the other is a true genealogical dataset from the province of Quebec. The fictive example is included in the package for educational purposes. Users can explore the functions using this example, which is not too big but still has interesting features. In order to illustrate what can be accomplished with the GENLIB package, we performed a complete description of the ascending genealogies available for the 140 individuals in the Quebec sample. These individuals come from various regions of Quebec and this information is also provided as a separate dataset within GENLIB. This sample has already been investigated to demonstrate the presence of population structure in genetic data, supported by genealogical information [28]. We then performed a simulation study to estimate the probability of sharing identical-by-descent (IBD) alleles and chromosomal segments of different lengths. Example source code for data analysis is provided in Additional file 3: Text S1.

For the Quebec sample, all participants gave informed written consent, provided family information necessary to reconstruct their genealogy using the BALSAC database, and agreed to the public distribution of their genealogy in coded form. The study was approved by the CHU Sainte-Justine Ethics Committee (reference number: 2684).

## Results

### Description of genealogical data using GENLIB

The Quebec sample provided with GENLIB includes 140 individuals (probands) sampled from seven regional or ethno-cultural populations. The regions represented are Montreal, Quebec City area, Saguenay, North Shore and Gaspesia in which we distinguish 3 different populations (French Canadians, Acadians and Loyalists). The whole genealogical corpus includes 41,523 individuals (20,773 males and 20,750 females). We identified 21,230 nuclear families, including 5,994 full-sibships of sizes varying from 2 to 14 individuals. These are ascending genealogies, i.e., they are reconstructed for each of the 140 probands separately by identifying his/her ancestors and then linked together. Hence, the number and sizes of sibships are underestimated.

**Figure 1:**
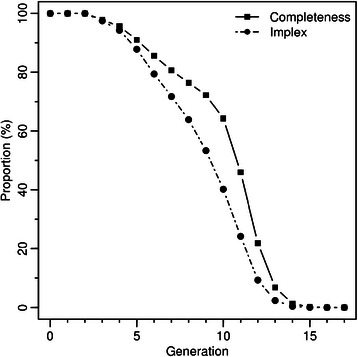
Completeness and implex indices for the Quebec genealogical corpus.

Including the initial generation (generation 0), this corpus is 18 generations deep. We identified 7,399 founders, i.e., individuals without parents in the BALSAC database, among all individuals in the genealogy. A few founders are in the second generation, meaning that some lineages stop after grandparents. However, more than 90% of the founders are in the 7th generation or higher. The mean genealogical depth is 9.4, indicating that on average genealogical lineages are 9.4 generations deep. Computed values of completeness and implex indices are shown on Figure 1. From the initial generation to the second back in time we observe that the whole genealogical corpus is known (completeness equals 100%) and its size grows exponentially from a generation to another (implex also equals 100%). From the third generation, the completeness and implex indices drop below 100% because pedigree collapsing starts and some information is missing. This means that 1) some lineages stop at the second generation, as noted earlier by the presence of founders in that generation, and 2) at least one individual has related ancestors in the first 3 generations, as indicated by an implex values slightly smaller than the completeness value. We also note that the implex index decreases faster than the completeness and that the gap between these two indices is the largest at the 10th generation.

**Figure 2:**
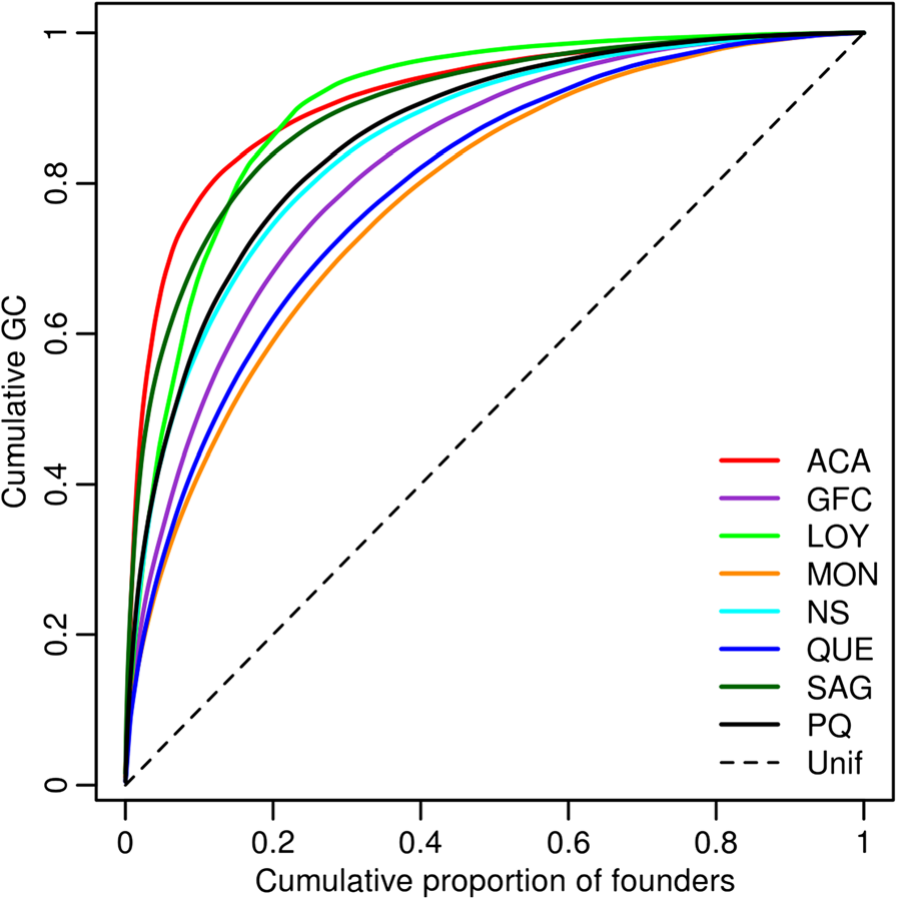
Cumulative genetic contribution of founders for each population. Plot of the cumulative distribution of genetic contributions of founders for each population in relation to the cumulative proportion of contributing founders, sorted in decreasing order of their genetic contribution. The dashed line presents the hypothetical situation in which all founders of a population contribute equally to the gene pool. *ACA* Acadians, *GFC* Gaspesian French Canadians, *LOY* Loyalists, *MON* Montreal, *NS* North Shore, *QUE* Quebec City area, *SAG* Saguenay, *PQ* Whole sample from the Province of Quebec, *Unif* Uniform distribution.

Figure 2 presents the relationship between the cumulative proportion of the genetic contribution of founders and the cumulative proportion of contributing founders. We observe that the genetic contribution of founders varies across populations with values quite far from the linear relationship resulting from the theoretical (unrealistic) uniform genetic contribution that would be obtained if every founder had an equal contribution to the gene pool. The sample from the Acadian sub-population shows the most uneven contribution of founders as 2.3% (n = 49) of their founders contribute to 50% of the gene pool. In comparison, the sample from Montreal has 14.4% (n = 794) of its founders contributing to 50% of the genetic pool. The larger contribution of some founders is explained by their differential demographic history, as well as the one from their descendants, in addition to their time of arrival in Quebec. Indeed it was previously observed that kinship coefficients vary among these populations (see Figure four from [28]). The highest inbreeding coefficient among all individuals in the genealogy (0.17) is observed for a proband from the Loyalist population. The parents of this individual are double first cousins, his grand-parents are double second cousins and his great-grand-parents are also first cousins (Additional file 1: Figure S1). Within the whole genealogy, 3.7% (n = 1549) of people have inbreeding coefficients greater than 1/64 corresponding to parents that are second cousins.

**Figure 3:**
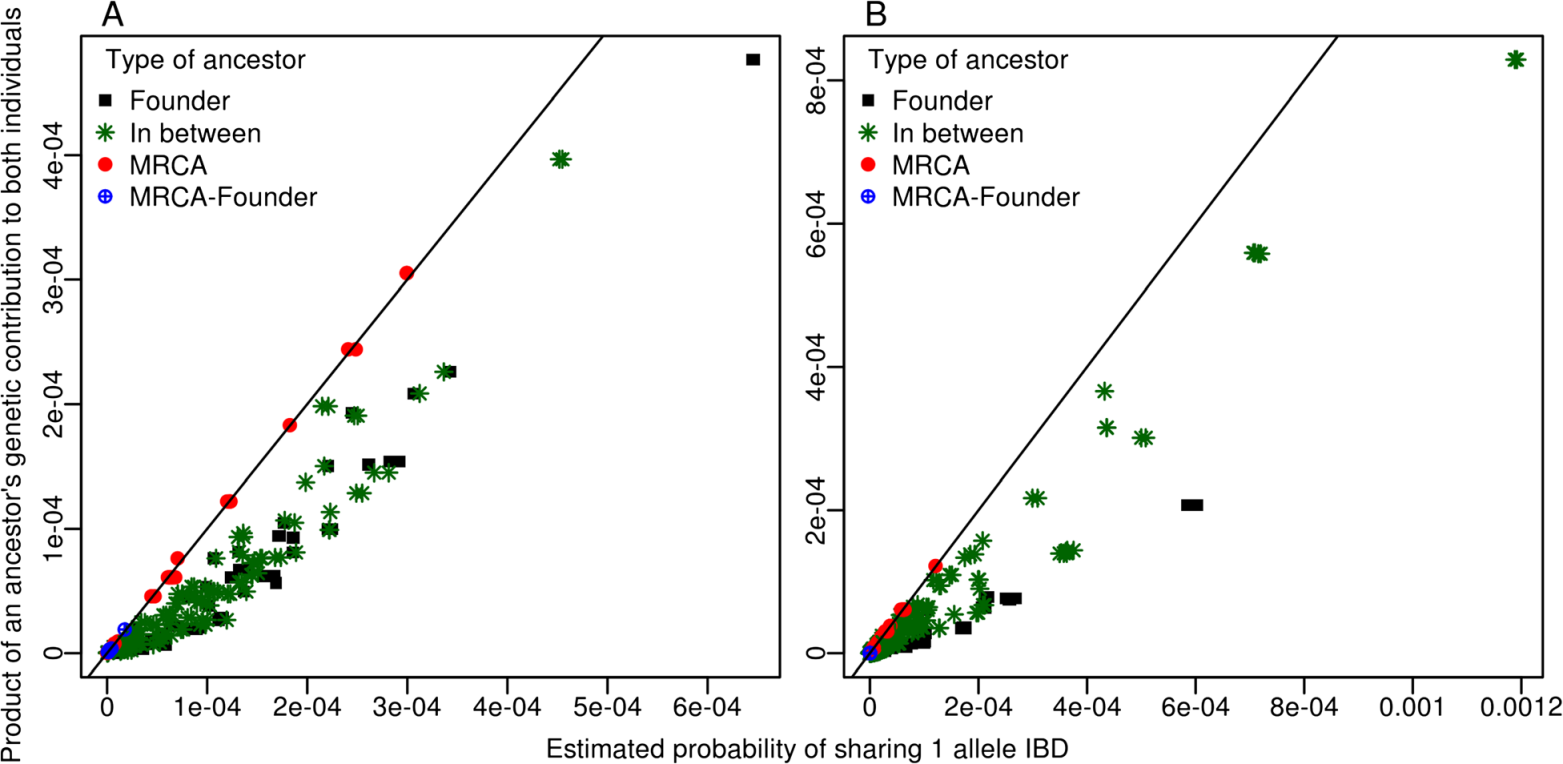
Estimated probabilities of sharing one allele IBD versus ancestors’ genetic contributions. Plots of estimated probabilities of sharing one allele identical-by-descent (IBD) from a specific ancestor relative to the product of the genetic contributions of that ancestor to each of the two individuals from **A)** the Acadian population and **B)** the Saguenay population. Probabilities that the two individuals share one allele IBD were estimated from 10,000,000 gene-dropping simulations for each shared ancestor. Ancestors are divided into four categories depending on whether they are founders, most recent common ancestors (MRCA), both (MRCA-Founder) or neither of the two (In between). The black line is the identity line, i.e. y = x.

The founders with the highest genetic contribution are not the ones with the highest coverage, i.e., linked to the maximum number of probands, because the genetic contribution is higher when an ancestor is closer while coverage increases as an individual is more distant. The high coverage individuals are all founders except one (who is the son of two of these high coverage founders) and each is linked to the same 121 probands out of all 140 probands in the data. The probands not covered by these prolific ancestors are mainly descendants of Loyalists, who arrived in Quebec after the American War of Independence, had little intermarriage with the surrounding French Catholic population and had less complete genealogical data. For distant ancestors, the genetic contribution is correlated with the number of occurrences of an ancestor within an individual’s genealogy, in other words how many different, but not necessarily mutually exclusive, paths link an ancestor to a descendant. The number of occurrences is maximal for a proband from Saguenay and some of its ancestors. Four founders and two of their children are linked 176 times trough different paths to this proband. These four founders also have the maximal genetic contribution in this genealogical corpus. All probands with ancestors occurring more than 60 times in their genealogies come from Saguenay and North Shore. Moreover, even if probands from the Acadian population have higher kinship values than those from the North Shore and Saguenay [28], we observe that North Shore and Saguenay are two populations showing an accumulation of distant common ancestors with high numbers of occurrences in the genealogy.

### Gene-dropping simulations using GENLIB

We performed gene-dropping simulations to evaluate the odds of IBD sharing for two pairs of distantly related individuals. We selected individuals that had similar kinship coefficients but different patterns of genealogical links (e.g., differences in the number of common ancestors and distances to these ancestors), so that patterns of IBD sharing are expected to differ between the pairs. For each pair of individuals, we identified all common ancestors and used these common ancestors as starting point for allele dropping simulations. We performed 10,000,000 simulations for each common ancestor and calculated the probability of IBD sharing as the proportion of simulations where at least one allele was shared. One pair of individuals is from the Saguenay population with a kinship coefficient of 0.0051 and shares 1628 common ancestors. The other pair of individuals is from the Acadian population with a kinship coefficient of 0.0044 and shares 261 common ancestors. Realized IBD sharing and genealogical measures have previously been compared between samples from the Saguenay and Acadian populations, indicating overall higher levels of IBD sharing in the Acadians in agreement with the observed smaller number of closer common ancestors [31]. Our goal here was to compare IBD sharing at distant kinship levels that are similar but arise from very different genealogical links, such as those present in the Acadian and Saguenay genealogical structures.

One advantage of gene-dropping simulations is that they give a more accurate picture of an ancestor’s impact on the genome of two individuals than the genetic contribution does. As can be seen on Figure 3, estimated probabilities of sharing one allele IBD are either perfectly correlated with the product of the two genetic contributions, either greater, depending on the ancestor from whom this allele was inherited (Additional file 2: Figure S2 illustrates the different types of ancestors). The odds of sharing an allele IBD depend on the number of meioses separating two individuals through a particular common ancestor and also on all the possible paths linking those two through this common ancestor. When we consider MRCAs, the product of an ancestor’s genetic contributions to the two probands is equal to the probability that the probands share one allele IBD because the paths from a proband to the MRCA and from the MRCA to the other proband are completely distinct. Otherwise, for non-MRCA ancestors (either founders or those in between founders and MRCA), the product of genetic contributions from that ancestor to both probands underestimates the probability of sharing an allele IBD. The more distantly linked two individuals are, the less likely they are to share an allele IBD and their variance for the number of IBD sharing occurrences across simulation replicates is also lower [41,42].

**Figure 4:**
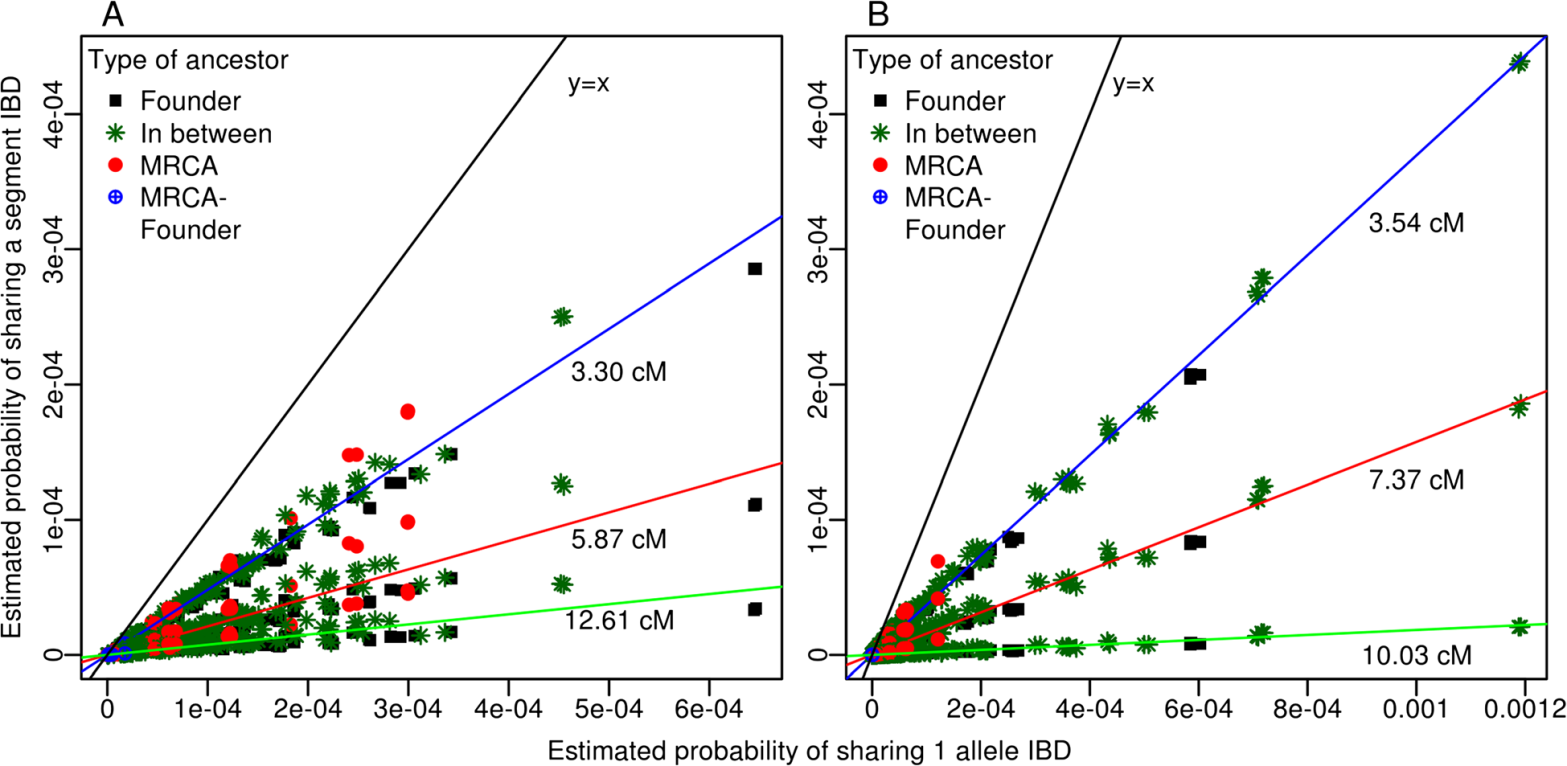
Estimated probabilities of IBD sharing for a segment versus one allele. Plots of estimated probabilities of sharing one segment identical-by-descent (IBD) from a specific ancestor relative to the estimated probabilities to share one allele IBD from that same ancestor for a pair of individuals from **A)** the Acadian population and **B)** the Saguenay population. Probabilities that the two individuals share one allele or one segment IBD were estimated from 10,000,000 (for allele sharing) or 100,000,000 (for segment sharing) simulations for each common ancestor. Ancestors are divided in four categories depending on whether they are founders, most recent common ancestors (MRCA), both (MRCA-Founder) or neither of the two (In between). The solid black line is the identity line and colored lines are simple regression lines between IBD sharing of a segment and IBD sharing of an allele. Three different segment lengths are considered and shown for A: 3.30, 5.87 and 12.61 cM and for B 3.54, 7.37 and 10.03 cM (Table 3).

We extended the simulation functionalities of GENLIB to simulate the transmission of chromosomal segments for each pair of individuals. Using IBD sharing previously inferred from genome-wide genotype data available on the 140 individuals [31], we selected multiple segments shared IBD by the two pairs of individuals described above. We used these observed segments to select the lengths of the segments to be simulated. This second set of simulations includes a probability that the segment undergoes recombination. For all segments we considered recombination rates based on sex-specific recombination maps [43] (see Table 3). We performed 100,000,000 simulations for each common ancestor. We were interested in the odds that the segment is transmitted intact from a common ancestor to both members of the pair, resulting in a segment shared IBD.

**Table 3:** **Selected segments shared IBD by two pairs of individuals**

**Individual IDs**	**Chr**	**Length (cM)**	**Length male recombination map (cM)**	**Length female recombination map (cM)**
408868,	1	3.5416	3.0829	4.2469
409033	5	7.3682	5.0353	9.0442
Acadian	8	10.0267	4.0278	14.9491
302710,	3	3.3035	2.2441	4.4612
302711	7	5.8712	4.0323	8.4025
Saguenay	1	12.6098	7.1124	17.3889

The results of the segment-dropping simulations were concordant with expectations. Indeed we observed that the probability of sharing a segment IBD decreases compared to the probability of sharing only one allele IBD with the increasing length of segments (Figure 4). Linear regressions for each segment length show how estimated probability of sharing a segment IBD decreases with an increasing recombination rate. Our ability to discriminate which ancestors could have contributed IBD segments is also facilitated with longer segments. Using a threshold on the probability of IBD sharing of 1.00E-08, corresponding to the 10th percentile of the overall probability distribution, we found that we could eliminate proportionally more potential ancestors unlikely to have transmitted a segment for the pair of individuals from Saguenay compared to the Acadians for similar segment lengths. For the smallest segment (~3.4 cM) we can exclude, respectively for the two Acadians and the two Saguenay individuals, 0 (0.0%) and 77 (4.7%) ancestors, for the midsize (~6.6 cM), 6 (2.3%) and 154 (9.5%) ancestors and for the longest segment size (~11.3 cM), 20 (7.7%) and 528 (32.4%) ancestors. As expected, segment length plays an important role since the longer the segment is, the higher the recombination chances are. Another important factor is the length of inheritance paths. Each time a segment is transmitted, it is subject to recombination so the longer a path between two individuals through an ancestor is, the higher the odds of recombination are.

## Discussion

In this paper, we presented GENLIB, an R package for the analysis of complex genealogical data. GENLIB can handle large extended human pedigrees, extended genealogical data from human founder populations or extended animal pedigrees. We were able to read and describe a fictive pedigree with a size over 10,000,000 and a depth of 58 generations. Some memory limits may be encountered, depending on the computer used, when trying to perform calculations (e.g., kinship) for a large number of individuals at the same time in complex genealogies, but these issues can easily be fixed by performing calculations in batches instead.

GENLIB is designed to be easily accessible and used by researchers familiar or not with genealogical information. The only requirement is a basic knowledge of the R environment. GENLIB includes functions to describe genealogies, to compute different genealogical indices and to perform simulations based on genealogical relationships. Given the flexibility of the R environment, users can also create their own functions to further describe, summarize and present (e.g., graphs) GENLIB results.

We used genealogies from the Quebec founder population to illustrate the utility of GENLIB to describe and analyze genealogical data. In addition, we performed simulations to track the transmission of alleles or chromosomal segments through the genealogy. Allele-or segment-dropping simulations that consider the complete genealogical structure can help to predict the risk of sharing disease mutations introduced by founders, or to identify ancestral sources of variation in a trait of interest [15,44]. In our simulations, we highlighted the fact that specific population characteristics, such as the number of shared ancestors and the distance to these shared ancestors, can help discriminate among founders more or less likely to have transmitted segments IBD. The fact that individuals from Saguenay share more common ancestors that are more distant on average compared to Acadians explains, in part, why, for similar kinship values, we can observe quite different IBD segment distributions. Gene-dropping simulations can also be used to predict the risk of future loss of genes contributed by the various founders. In addition, distributions of IBD probabilities for any individuals can be generated through simulations.

We plan to continue to improve existing functions, for example by adding more flexible options for individual fitness in the simulations. We also plan to extend the range of functions available. Examples of planned additions include implementing more data importation and graphical functionalities, incorporating information about probands and ancestors, such as birth places and dates, implementing a function to flag unrelated components (subpedigrees) within a kindred, and implementing statistical tests to compare kinship and inbreeding distributions between different groups. Another planned addition is to include an option in the segment-dropping simulations to recover the length of transmitted segments instead of only its status (fully transmitted or not). With its flexibility, GENLIB provides an important contribution towards an improved and optimal way to combine information coming from genealogies and genetic material. These two sources of data are complementary and using them together could improve genetic data analysis in founder populations, for example in the context of estimating relatedness, imputing genomic data or haplotyping [28,45,46].

## Conclusions

The GENLIB R package was developed to facilitate a broader and more extensive use of available genealogical data from founder populations across the world. The package provides a user friendly and flexible environment to analyze genealogical data, allowing a more efficient and easier integration of different types of data and analytical methods and making it ideal for further developments. Using GENLIB simply requires a minimal knowledge of R and provides many functions to manage, describe and analyze genealogical data. Our description of a Quebec genealogical sample and our simulation study illustrated the use of GENLIB and further highlighted regional differences present in the population. We also provided insight on factors influencing the resulting IBD sharing in founder populations. GENLIB is an improvement over existing software in that it provides gene- and segment-dropping simulations and other new functionalities and in that it can be used within R, which is a computing environment familiar to many statistical geneticists. Hence, it is a highly valuable tool to researchers studying extended genealogies.

## Availability and requirements

**Project name:** GENLIB

**Project home page:** http://balsac.uqac.ca/

**Operating system(s):** Linux, Microsoft Windows

**Programming languages:** R, C++

**Other requirements:** the package depends on the R packages: kinship2, methods, bootstrap, Matrix, lattice, quadprog, foreach, parallel, doParallel, Rcpp

**License:** GNUGPL

**Any restrictions to use by non-academics:** None

## Additional files

Additional file 1: Figure S1. Genealogy of a highly inbred individual. Genealogical tree for one individual from the Loyalist population. Lineages are cut as soon as unrelated individuals are found.
Additional file 2: Figure S2. Genealogical example showing different types of common ancestors. Two probands can share either 1) most recent common ancestors (MRCA), 2) MRCA which are also founders (MRCA-Founder), 3) ancestors between MRCA and founders called “In between” or 4) founders.
Additional file 3:
**Text S1.** Example source code for the description and analysis of the genealogical data presented.
