# Supplementary figures and images for: GENLIB: an R package for the analysis of genealogical data
Source: BMC Bioinformatics. 2015 May 15;16:160. doi: 10.1186/s12859-015-0581-5 (PMC4431039; doi:10.1186/s12859-015-0581-5)

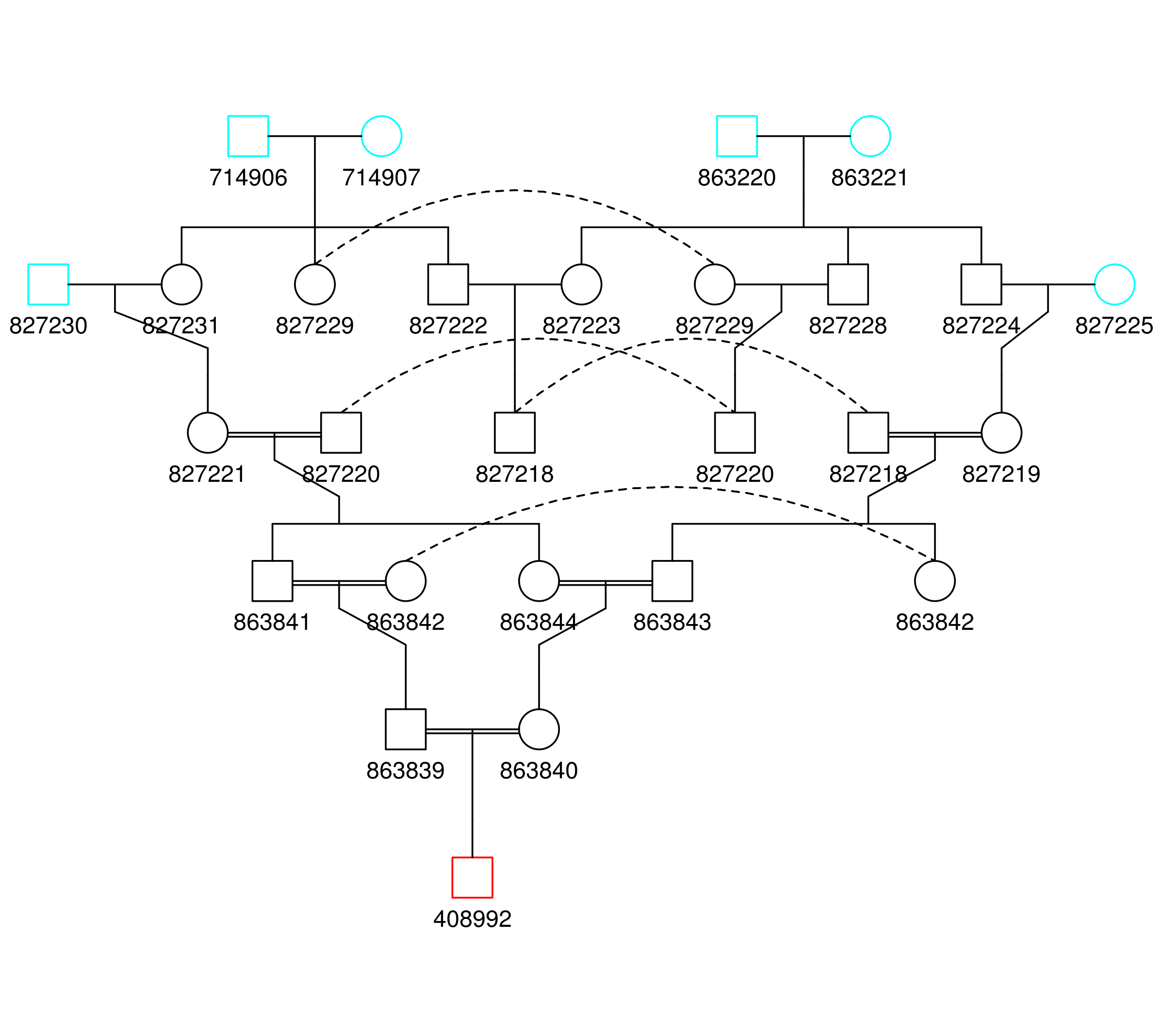

Supplement: Additional file 1: Figure S1. — Genealogy of a highly inbred individual. Genealogical tree for one individual from the Loyalist population. Lineages are cut as soon as unrelated individuals are found. [file 12859_2015_581_MOESM1_ESM.png]

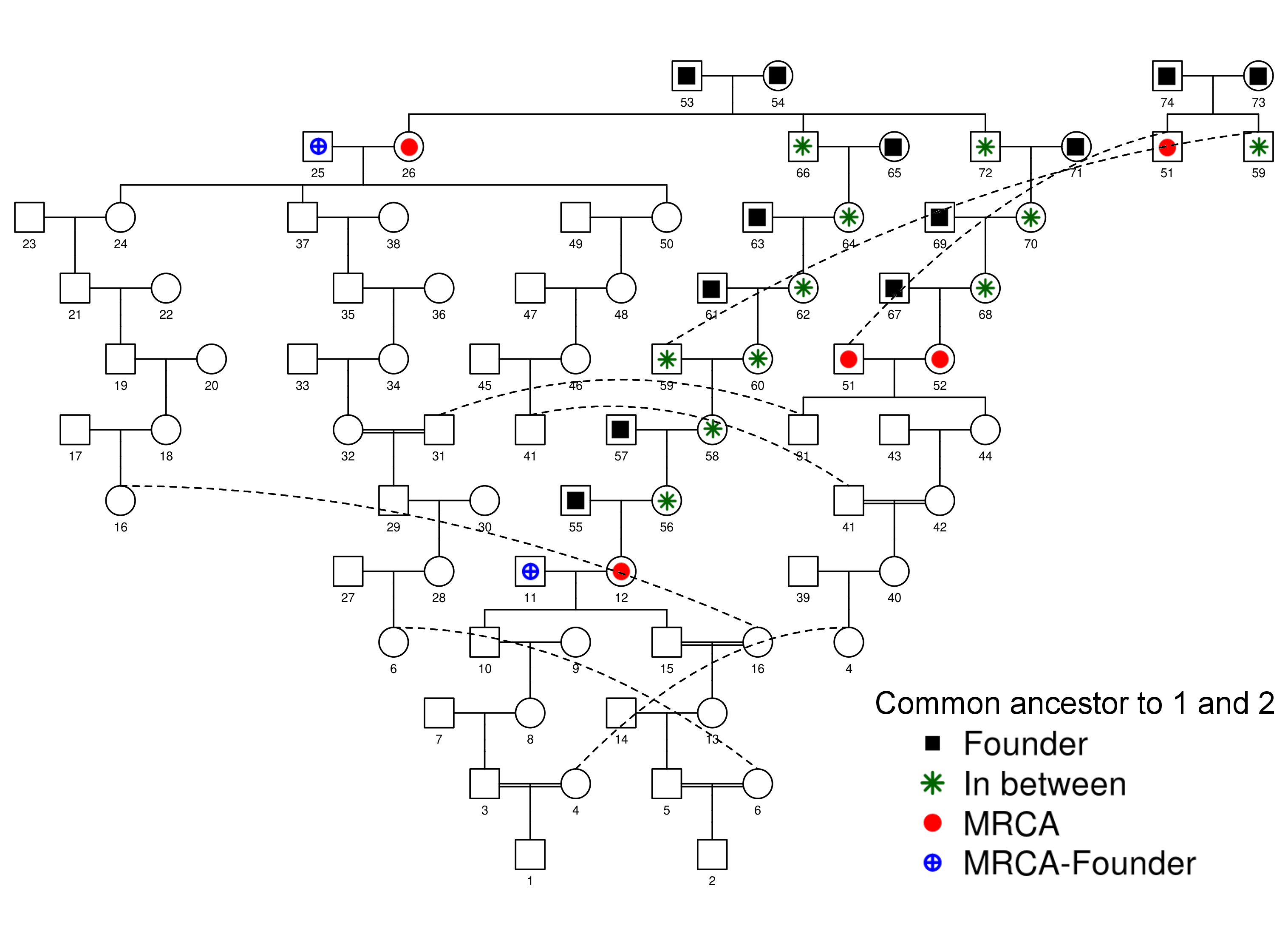

Supplement: Additional file 2: Figure S2. — Genealogical example showing different types of common ancestors. Two probands can share either 1) most recent common ancestors (MRCA), 2) MRCA which are also founders (MRCA-Founder), 3) ancestors between MRCA and founders called “In between” or 4) founders. [file 12859_2015_581_MOESM2_ESM.png]
